# A risk scoring system for advanced colorectal neoplasia in high-risk participants to improve current colorectal cancer screening in Tianjin, China

**DOI:** 10.1186/s12876-022-02563-9

**Published:** 2022-11-17

**Authors:** Zhen Yuan, Shuyuan Wang, Zhaoce Liu, Yanfei Liu, Yuqi Wang, Youkui Han, Weifeng Gao, Xinyu Liu, Hongzhou Li, Qinghuai Zhang, Hong Ma, Junying Wang, Xiaomeng Wei, Xipeng Zhang, Wei Cui, Chunze Zhang

**Affiliations:** 1 School of Medicine, Nankai University, Tianjin, China; 2 Department of Colorectal Surgery, Tianjin Union Medical Center, Tianjin, China; 3 School of Integrative Medicine, Tianjin University of Traditional Chinese Medicine, Tianjin, China; 4 Department of General Surgery, Tianjin Union Medical Center, Tianjin, China; 5 Tianjin Medical University, Tianjin, China; 6 Department of Endoscopy, Tianjin Union Medical Center, Tianjin, China; 7 The Institute of Translational Medicine, Tianjin Union Medical Center of Nankai University, Tianjin, China; 8 Tianjin Institute of Coloproctology, Tianjin, China; 9 Nursing Department, Tianjin Union Medical Center, Tianjin, China; 10 Hospital Infection Management Division, Tianjin Union Medical Center, Tianjin, China; 11 School of Mathematical Sciences and LPMC, Nankai University, Tianjin, China

**Keywords:** Colorectal cancer, Advanced colorectal neoplasia, Screening, Risk scoring systems

## Abstract

**Background:**

Given the limited effectiveness of the current Chinese colorectal cancer (CRC) screening procedure, adherence to colonoscopy remains low. We aim to develop and validate a scoring system based on individuals who were identified as having a high risk in initial CRC screening to achieve more efficient risk stratification and improve adherence to colonoscopy.

**Methods:**

A total of 29,504 screening participants with positive High-Risk Factor Questionnaire (HRFQ) or faecal immunochemical test (FIT) who underwent colonoscopy in Tianjin from 2012–2020 were enrolled in this study. Binary regression analysis was used to evaluate the association between risk factors and advanced colorectal neoplasia. Internal validation was also used to assess the performance of the scoring system.

**Results:**

Male sex, older age (age ≥ 50 years), high body mass index (BMI ≥ 28 kg/m^2^), current or past smoking and weekly alcohol intake were identified as risk factors for advanced colorectal neoplasm. The odds ratios (ORs) for significant variables were applied to construct the risk score ranging from 0–11: LR, low risk (score 0–3); MR, moderate risk (score 4–6); and HR, high risk (score 7–11). Compared with subjects with LR, those with MR and HR had ORs of 2.47 (95% confidence interval, 2.09–2.93) and 4.59 (95% confidence interval, 3.86–5.44), respectively. The scoring model showed an outstanding discriminatory capacity with a c-statistic of 0.64 (95% confidence interval, 0.63–0.65).

**Conclusions:**

Our results showed that the established scoring system could identify very high-risk populations with colorectal neoplasia. Combining this risk score with current Chinese screening methods may improve the effectiveness of CRC screening and adherence to colonoscopy.

## Background

Colorectal cancer (CRC) is the third most common cancer and second most lethal cancer worldwide [1]. In terms of cancer incidence and mortality in China, in 2020, there were 0.56 million new cases of CRC, and CRC was responsible for 0.29 million deaths [2]. Generally, the development of CRC is a multistage and slow process occurring over several decades, with colorectal neoplasia (CRN) and advanced colorectal neoplasm (ACN) representing key steps in progression to CRC [3]. If detected and treated in the early stages, the prognosis is favourable, with a five-year survival rate reaching 90% [4].

Current guidelines recommend that individuals over 45 years old should undergo CRC screening to reduce CRC-related incidence and mortality [5–7]. Many countries and regions, such as Korea, America and China, have implemented CRC screening programs [8, 9]. The faecal occult blood test (FOBT)/faecal immunochemical test (FIT) and colonoscopy are the two most common screening tools [10]. However, extensive screening programs with colonoscopy have not been implemented in some countries due to resource and staffing constraints [11, 12]. FIT is relatively simple and inexpensive, but its sensitivity and specificity are limited, which may lead to missed diagnosis in high-risk persons [13]. Therefore, a risk stratification strategy is needed for persons undergoing CRC screening to make the most of the limited resources and improve the efficiency of screening.

Multiple risk scoring systems have been introduced in various countries for CRC screening [14–16]. In China, the high-risk factor questionnaire (HRFQ) was proposed for risk stratification and was based on the CRC screening project in two counties of Zhejiang Province [17]. Subsequently, the combination of the HRFQ and FIT has been adapted by the Chinese Ministry of Health since 2006 [18] and implemented in many cities across China, including Shanghai, Guangzhou, and Tianjin, and significant results have been achieved [19–22]. In this screening procedure, subjects with positive FIT or HRFQ were classified as high risk and recommended to undergo colonoscopy [21]. Nevertheless, previous studies have found that adherence to further colonoscopy follow-up was generally low, and less than 30% of high-risk participants underwent colonoscopy [20, 23]. The current risk stratification has a high sensitivity but a high false positive rate, leading to unnecessary colonoscopy, which results in doubts about the effectiveness of screening and decreases compliance [24]. In addition, some of the latest well-documented risk factors, such as smoking status, alcohol intake and BMI, were not included in the current HRFQ and may affect the discriminatory ability of the screening procedure [25].

In this study, we developed a risk scoring model for predicting colorectal lesions based on the high-risk population in the Tianjin CRC screening programme from 2012–2020. Our findings may help to improve the commonly used risk stratification methods, promote adherence to colonoscopy and increase the efficiency of CRC screening in China.

## Methods

### Study setting

This study was conducted at Tianjin Union Medical Center as a part of the Tianjin CRC screening programme. The Tianjin CRC screening programme was established in 2012 by the government, and it already provided free CRC screening for more than 6 million residents aged > 40 years in Tianjin from 2012–2020. A two-step method similar to that of Jiashan County [18, 26] was applied in the CRC screening programme, in which HRFQ and FIT were used for initial screening, and subjects with any positive HRFQ or FIT were recommended to undergo colonoscopy screening. The population of this study was a subset of the screening programme, and this study was approved by the Ethics Committee of Tianjin Union Medical Center.

### Study participants

This study retrospectively analysed the data obtained from the prospective screening programme. The inclusion criteria included the following: (1) Participants defined as high-risk in initial screening, with positive FIT (ABON, China) or (and) positive-HRFQ. Positive HRFQ was defined as subjects meeting any of following conditions: (a) history of any cancer or colorectal polyps; (b) history of CRC in first-degree relatives; (c) history of two or more of the following symptoms: chronic diarrhoea, chronic constipation, serious unhappy life events such as death among first-degree relatives, mucus or bloody stool, chronic appendicitis or appendectomy, chronic cholecystitis or cholecystectomy; (2) those who underwent subsequent colonoscopy after being identified as high risk by FIT or (and) HRFQ; and (3) those who wished to participate and signed informed consent form. Subjects with a history of CRC were excluded from this study.

### Colonoscopy procedures

All endoscopic examinations were performed in the hospital by experienced endoscopists who had at least 5 years of experience and were all board certified to perform endoscopy. All abnormal findings were confirmed by expert gastrointestinal pathologists following up-to-date clinical guidelines. Only high-quality colonoscopies were included, with adequate bowel preparation, photo documentation of caecal landmarks, and a withdrawal time > 6 min.

Colorectal lesions were classified as ulcerative colitis or Crohn’s disease, chronic inflammatory bowel disease, ulcer, adenoma, and CRC [20]. ACN was defined as CRC or advanced adenoma ≥ 10 mm in diameter or with villous components or high-grade dysplasia. CRN was defined as cancer or any adenoma.

### Measurements and definitions

In this study, smoking status was categorized as never smoker and current or past smoker. Alcohol consumption was categorized as never or occasional alcohol intake and weekly alcohol intake. Regular exercise was defined as at least 30 min of exercise more than once weekly over the last year; otherwise, it was classified as ‘physical inactivity’. Obesity was defined as BMI ≥ 28 kg/m^2^ according to Chinese criteria [27]. Educational level was categorized as low (primary education or below), intermediate (secondary education, high education or lower vocational education) and high (higher vocational education, university or above).

### Development of risk scores

The association between the prevalence of ACN and clinical risk factors was assessed by univariate and multivariate logistic regression models. The risk factors examined included sex, age, body mass index (BMI), smoking status, alcohol intake and physical activity. All risk factors with *p* values less than 0.05 in univariate analysis were included in the binary logistic regression model for ACN. A scoring model for predicting ACN was developed based on the results of multivariate analysis, and each variable was assigned a weight using the respective adjusted OR rounded to the nearest integer. The final score of each participant was the sum of scores for each risk factor.

### Statistical analysis

All statistical analyses were performed using R software (V.4.1.2). The prevalence of CRN, ACN and CRC was calculated in participants stratified by clinical parameters and assessed with the chi-square test. The odds ratio (OR) with 95% confidence interval (95% CI), beta-confidence and standard error of each variable were calculated by a logistic regression model. The prevalence of ACN was evaluated according to each score, and then screened individuals were divided into three subgroups according to the final scores: “low risk (LR)”, “moderate risk (MR)” and “high risk (HR)”. A bootstrapping test with 1000 replicates was performed to internally validate the new scoring model. To evaluate the discriminatory capability of the model, a receiver operating characteristic (ROC) curve was plotted, and c-statistics were calculated. *P* values less than *0.05* were considered statistically significant.

## Results

### Characteristics of participants

**Fig. 1:**
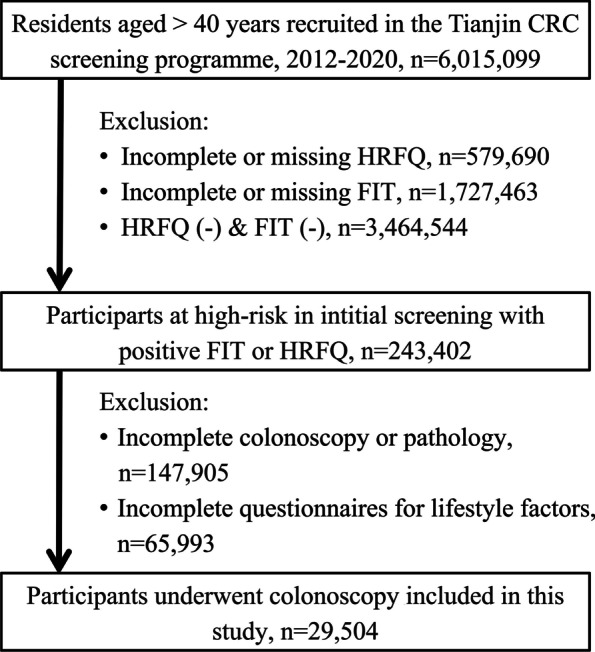
Flowchart depicting the process of patient inclusion. CRC, colorectal cancer

The population-based CRC screening process is shown in Fig. 1. The clinical parameters and colorectal lesions of the participants are summarized in Table 1. There were 29,504 high-risk participants enrolled in this study, with 20,299 (68.8%) having a positive FIT and 13,460 (45.6%) having positive HRFQ results. Of these, 14,677 (49.7%) individuals were found to have CN, including 2324 (7.9%) diagnosed with ACN. The average age of all subjects was 63.53 years (SD 7.47), 15,710 (53.2%) were female participants, 16.0% had a BMI greater than 28 kg/m^2^, and 24.4% were current or past smokers.

**Table 1:** Prevalence of colorectal neoplasia, advanced neoplasia, and colorectal cancer in the cohort according to risk factors

	**All subjects**	Colorectal neoplasia^*^		**Advanced neoplasia**		**Cancer**	
	(*n* = 29,504)	(*n* = 14,677)		(*n* = 2324)		(*n* = 462)	
	*n* (%)	Prevalence (%)	*p* value	Prevalence (%)	p value	Prevalence (%)	*p* value
**Sex, n (%)**							
Female	15,710 (53.2)	6557 (44.7)	< 0.001	897 (38.6)	< 0.001	220 (47.6)	0.017
Male	13,794 (46.8)	8120 (55.3)		1427 (61.4)		242 (52.4)	
**Age, y, mean ± SD**	63.53 (7.47)	64.55 (7.00)	< 0.001	66.05 (6.58)	< 0.001	67.62 (6.19)	< 0.001
**Age groups, y, n (%)**							
< 50	1295 (4.4)	379 (2.6)	< 0.001	30 (1.3)	< 0.001	2 (0.4)	< 0.001
50–60	7087 (24.0)	2985 (20.3)		332 (14.3)		49 (10.6)	
60–70	14,690 (49.8)	7691 (52.4)		1241 (53.4)		225 (48.7)	
≥ 70	6432 (21.8)	3622 (24.7)		721 (31.0)		186 (40.3)	
**BMI, kg/m**^**2**^**, mean ± SD**	24.95 (3.27)	25.19 (3.26)	< 0.001	25.28 (3.26)	< 0.001	25.24 (3.48)	0.052
**BMI groups, kg/m2, n (%)**							
< 28	24,769 (84.0)	12,084 (82.3)	< 0.001	1884 (81.1)	< 0.001	367 (79.4)	0.009
≥ 28	4735 (16.0)	2593 (17.7)		440 (18.9)		95 (20.6)	
**Smoking status, n (%)**							
Never	22,305 (75.6)	10,283 (70.1)	< 0.001	1552 (66.8)	< 0.001	354 (76.6)	0.644
Current or past^**^	7199 (24.4)	4394 (29.9)		772 (33.2)		108 (23.4)	
**Alcohol intake, n (%)**							
Never or occasional	26,170 (88.7)	12,534 (85.4)	< 0.001	1901 (81.8)	< 0.001	396 (85.7)	0.049
Weekly	3334 (11.3)	2143 (14.6)		423 (18.2)		66 (14.3)	
Regular activity^***^, n (%)							
Yes	13,460 (45.6)	7033 (47.9)	< 0.001	1190 (51.2)	< 0.001	260 (56.3)	< 0.001
No	16,044 (54.4)	7644 (52.1)		1134 (48.8)		202 (43.7)	
**Educational level, n (%)**							
Low	6671 (22.6)	3254 (22.2)	0.199	466 (20.1)	0.007	86 (18.6)	0.1
Intermediate	19,535 (66.2)	9771 (66.6)		1581 (68.0)		318 (68.8)	
High	3298 (11.2)	1652 (11.3)		277 (11.9)		58 (12.6)	
**Marital status, n (%)**							
Married or living with significant other	28,215 (95.6)	14,047 (95.7)	0.541	2222 (95.6)	1	441 (95.5)	0.942
Living alone	1289 (4.4)	630 (4.3)		102 (4.4)		21 (4.5)	
**FIT test (%)**							
Negative	9205 (31.2)	4392 (29.9)	< 0.001	466 (20.1)	< 0.001	104 (22.5)	< 0.001
Positive	20,299 (68.8)	10,285 (70.1)		1858 (79.9)		358 (77.5)	
**HRFQ (%)**							
Negative	16,044 (54.4)	8141 (55.5)	< 0.001	1503 (64.7)	< 0.001	281 (60.8)	0.006
Positive	13,460 (45.6)	6536 (44.5)		821 (35.3)		181 (39.2)	

*BMI* Body mass index, *FIT* Faecal immunochemical test

^*^ Colorectal neoplasia includes adenoma, advanced neoplasia and cancer. Advanced neoplasia includes colorectal cancer, any colorectal adenoma that has a size of ≥ 10 mm in diameter, high-grade dysplasia, villous or tubulovillous histologic characteristics, or any combination thereof

^**^Current smoking denotes ≥ 1 pack of cigarettes/week

^***^Regular activity denotes at least 30 min of exercise more than once weekly over the last year

### Univariate and multivariate predictors of ACN

The association between the prevalence of ACN and risk factors evaluated by univariate and multivariate analyses is shown in Table 2. From multivariate analysis adjusted for educational level and marital status, each 10-year increase in age from 50 years onwards (OR = 2.14–5.52), male sex (OR = 1.63, 95% CI = 1.49–1.79), BMI ≥ 28 kg/m^2^ (OR = 1.20, 95% CI = 1.08–1.33), ever smoking (OR = 1.17, 95% CI = 1.06–1.29) and weekly alcohol intake (OR = 1.22, 95% CI = 1.08–1.38) were significantly correlated with the presence of ACN, while physical inactivity showed no correlation with ACN in univariate or multivariate analysis.

**Table 2:** Univariate and multivariate predictors of colorectal advanced neoplasia in this cohort

Unadjusted			Adjusted			
	OR (95% CI)	*p* value	β coefficient	SE	AOR (95% CI)	*p* value
**Sex**						
Female	Reference					
Male	1.91 (1.75,2.08)	< 0.001	0.490	0.048	1.63 (1.49,1.79)	< 0.001
**Age, years**						
< 50	Reference					
50–60	2.07 (1.42,3.03)	< 0.001	0.759	0.180	2.14 (1.50,3.04)	< 0.001
60–70	3.89 (2.7,5.61)	< 0.001	1.399	0.174	4.05 (2.88,5.70)	< 0.001
≥ 70	5.32 (3.68,7.71)	< 0.001	1.709	1.710	5.52 (3.91,7.81)	< 0.001
**BMI, kg/m**^**2**^						
< 28	Reference					
≥ 28	1.24 (1.12,1.39)	< 0.001	0.179	0.053	1.20 (1.08,1.33)	< 0.001
**Smoking status**						
Never	Reference					
Current or past	1.61 (1.47,1.76)	0.002	0.157	0.052	1.17 (1.06,1.29)	0.002
**Alcohol intake**						
Never or occasional	Reference					
Weekly	1.86 (1.66,2.08)	0.001	0.201	0.063	1.22 (1.08,1.38)	0.001
**Regular activity**						
Yes	Reference					
No	0.78 (0.72,0.85)	0.731	0.015	0.043	1.01 (0.93,1.10)	0.732

### Development of the risk score

A scoring model for predicting ACN was developed, and points were assigned to relative risk factors as follows (Table 3): male sex (2), female sex (0), age < 50 years (0), age 50–60 years (2), age 60–70 years (4), age ≥ 70 years (6), BMI ≥ 28 kg/m^2^ (1), BMI < 28 kg/m^2^ (0), current or past smoking (1), never smoking (0), weekly alcohol intake (1), and never or occasional alcohol intake (0). The scoring system ranged from 0–11, and the points were weighted according to ORs from the multivariate analysis rounding to the nearest whole number. The number of participants with different scores and the prevalence of ACN by scores are presented in Table 4.

**Table 3:** Scoring model for the prediction of risk for advanced colorectal neoplasia

Risk factor	Criteria	Points
Sex	Female	0
	Male	2
Age, years	< 50	0
	50–60	2
	60–70	4
	≥ 70	6
BMI, kg/m^2^	< 28	0
	≥ 28	1
Smoking status	Never	0
	Current or past	1
Alcohol intake	Never or occasional	0
	Weekly	1

**Table 4:** Prevalence of advanced colorectal neoplasia by risk tier and risk score

Score	Subjects, n (%)	Subjects with ACN, n (%)	Risk tier	Proportion of individuals with ACN	95% CI	Relative risk (95% CI)
0	585	9 (1.5)	Low risk			
1	53	1 (1.9)	0–3	3.0%	2.6–3.5	Reference
2	3789	122 (3.2)		(158/5267)		
3	840	26 (3.1)				
4	7495	395 (5.3)	Moderate risk	7.1%		
5	2690	198 (7.4)	4–6	(1129/15888)	6.7–7.5	2.47 (2.09–2.93)
6	5703	536 (9.4)				
7	3147	345 (11.0)	High risk			
8	3092	424 (13.7)	7–11	12.4%		
9	1373	168 (12.2)		(1037/8349)	11.7–13.1	4.59 (3.86–5.44)
10	646	85 (13.2)				
11	91	15 (16.5)				
Total	29,504	2324 (7.9)				

### Discriminatory ability and reliability of the risk score

Subjects were divided into three risk tiers: LR, low risk (score 0–3); MR, moderate risk (score 4–6); and HR, high risk (score 7–11). The proportions of LR, MR and HR were 17.9% (5267/29504), 53.8% (15,888/29504) and 28.3% (8349/29504), respectively. Subjects with MR had a prevalence of ACN similar to the overall prevalence (7.1% vs. 7.9%). Compared with subjects with LR, those with MR and HR had ORs of 2.47 (2.09–2.93) and 4.59 (3.86–5.44), respectively (Table 4). Furthermore, internal validation showed a c-statistic of 0.64 (95% CI = 0.63–0.65) in the 1000 bootstrapped samples, which showed that it has a relatively stable risk-stratification ability.

## Discussion

In this study, we developed a scoring system for further risk stratification of a CRC high-risk population classified by the current screening procedure. The risk score derived from the logistic regression model included five well-recognized risk factors (sex, age, BMI, alcohol intake and smoking status) that were not included in the current screening tools. The scoring model performed well, and the ACN risk was found to be 2–sixfold higher in subjects with scores over 6 than in other individuals. Given the high false positive rate and low adherence to colonoscopy in participants, our findings have the potential to serve as a powerful complement to existing screening strategies and improve the effectiveness of screening programs.

After risk stratification by this scoring system, the prevalence of ACN in the high-risk group increased from 7.9% to 12.4%. Colonoscopy resources remain limited across China, and the application of this scoring system could take better advantage of these resources, which may help increase the discovery rate of colorectal lesions and help more patients with colorectal neoplasia diagnosed at an early stage. Integrating this scoring model with current screening methods could improve the effectiveness of screening programs, which will make populations more trusting of the results of risk stratification and more willing to receive colonoscopy tests.

Multiple scoring models for predicting ACN have been constructed in previous studies, containing common variables (sex, age, smoking status and family history) and subtle differences in other factors [14, 15, 28, 29]. Sekiguchi et al. proposed a scoring system for risk stratification in Japanese average-risk populations using five risk factors, including sex, age, family history, BMI and smoking history, which stratified screened subjects into three risk groups [14]. The most well-known Asia–Pacific Colorectal Screening score (APCS) also incorporated age, sex, family history and smoking to predict the risk for ACN, which was created based on the population from 11 countries in Asia [28], and the factors included in our scoring model were similar to those of previous studies. In addition, some well-recognized factors, such as family CRC history and personal cancer history, were not considered in our score, since they were included in the HRFQ and have been evaluated before.

The predictive ability of our score was consistent with that of previous studies; participants with higher scores had a significantly higher risk of colorectal neoplasia. The prevalence of ACN in our study was 12.2%, which was slightly higher than that in studies conducted in Japan (10.2%) [14] and the USA (9.0%) [16]. In addition, participants with high and moderate risk had a 4.59- and 2.40-fold higher risk of ACN, respectively, than those with low risk, which was consistent with the results of the APCS study [28]. The c-statistic of this score was 0.64, superior to that established by Liang et al. (c-statistic = 0.62) [30] and comparable to multiple previous scoring systems [14, 15].

Previous studies mainly focused on average-risk asymptomatic populations [14, 28], while we pioneered to focus on a relatively high-risk population according to HRFQ and FIT screening. In mainland China, nearly every large-scale CRC screening programme has adapted a two-step procedure, using HRFQ as the first step and colonoscopy as the second step [19–22]. Nevertheless, the HRFQ was implemented by the Chinese Ministry of Health in 2006, and some newly identified factors were not included in it [18]. Thus, it is of vital importance to develop a scoring system incorporating the latest well-recognized neoplasia-related risk factors to improve the current imperfect HRFQ. For instance, male sex and older age were found to be associated with ACN in various studies [28, 31, 32].

Individuals with overweight and obesity account for at least 11% of CRC cases in Europe, and each 1-kg/m^2^ increase in BMI confers an additional risk of CRC (HR = 1.03) [33, 34]. Lee et al. reported that the prevalence of colorectal polyps was 3.5 times higher in current smokers than in never smokers [35]. Alcohol intake is a well-documented risk factor for CRC, and a recent meta-analysis based on 8 large-scale studies revealed that heavy consumption was associated with an increased risk of CRC [36, 37]. Moreover, the latest study in *Lancet* reported that the leading risk factors contributing to global cancer burden were smoking, alcohol use and high BMI [38]. The scoring models involving the factors above have been implemented in many countries, such as Korea [15], Japan [14] and the USA [29], and incorporating these factors into Chinese official CRC screening programmes will improve the effectiveness of screening and increase adherence to colonoscopy.

There were several limitations to this study. First, although we have done our best to analyse all CRC-related factors, some potential related factors, such as diabetes [39], dietary habits and lifestyle [40], were still not considered since the information was not collected. Second, external validation of this risk score was not performed due to the lack of external data. Third, most indicators were self-reported by participants, which may introduce recall bias. Only subjects who underwent colonoscopy were involved in this study, which may cause selection bias. Finally, this score was developed based on participants in Tianjin, and generalizability to other settings or cities should be done with caution.

## Conclusions

In conclusion, we developed a scoring system for further risk stratification of a high-risk population classified by HRFQ and FIT that could identify very high-risk populations with colorectal neoplasia. Combining this risk score with current Chinese screening methods may improve the effectiveness of CRC screening and adherence to colonoscopy.

## Data Availability

The datasets analysed during the current study are not publicly available due to the data confidentiality requirements of Tianjin Health Commission but are available from the corresponding author on reasonable request.
